# Can canines alone be used for age estimation in Chinese individuals when applying the Kvaal method?

**DOI:** 10.1080/20961790.2020.1717029

**Published:** 2020-03-18

**Authors:** Mujia Li, Jiamin Zhao, Wenjie Chen, Xin Chen, Guang Chu, Teng Chen, Yucheng Guo

**Affiliations:** a Key Laboratory of Shaanxi Province for Craniofacial Precision Medicine Research, College of Stomatology, Xi’an Jiaotong University, Xi’an, China; b Department of Orthodontics, Stomatological Hospital of Xi’an Jiaotong University, Xi’an, China; c College of Medicine and Forensics, Xi’an Jiaotong University Health Science Center, Xi’an, China

**Keywords:** Forensic sciences, forensic odontology, age estimation, canine, secondary dentine, Kvaal method, Chinese subjects

## Abstract

Due to the secondary dentin formation, the dental pulp undergoes changes in shape throughout life. Based on this phenomenon, the Kvaal method has been applied to various populations for age estimation, and its usefulness has been verified. When applying the Kvaal method to Chinese subjects, we observed a relatively strong correlation between mandibular canines and age. This study notes the correlation between canines and chronological age and is the first to identify which canine is most closely related to chronological age. In addition, a new, simpler formula is determined based on canines according to Kvaal’s methodology. The radiographs of 360 individuals from northern China were selected, from which the widths and lengths of the pulp from four canines were measured according to the Kvaal method. Next, inter- and intra-observer reliabilities were analyzed in order to assess the repeatability of these measurements. The correlation between measurements and age was examined, and Chinese-specific age estimation formulae were derived. The results revealed that the ratios from the left maxillary canine exhibited the strongest correlation with age compared to the other canines, whereas the left mandibular canine showed the weakest correlation, which may contribute to the overall poor correlation of mandibular canines with age. What’s more, the formula derived from the left maxillary canine in this study displayed the highest coefficients of determination, and the formula derived from all canines showed the lowest residuals. Both of these formulae performed better than the Chinese-specific formula derived from six different types of teeth in our previous study, which had formerly possessed the highest coefficients of determination and the lowest residuals. Thus, we concluded that canines do play an important role in age estimation in the Chinese population, and the correlation between maxillary canines and chronological age is stronger than that of mandibular canines, although no distinct trend as to which side is better correlated with age was established. Going forward, we recommend the analysis of additional samples from different geographical regions and populations to further verify the importance of canines in age estimation.

## Introduction

Age estimation is important in forensic science since this information may be utilized to resolve issues relating to refugee dynamics, competitive sports and court cases [1–4]. Teeth have a certain advantage in age estimation because they are the strongest and longest-lasting physical structures in the body, and are protected by facial tissue [5,6].

Various methods based on physiological changes in teeth have been proposed for age estimation [7–11]. Mineralization of third molars was once used to determine whether a person is over 18 years of age, but third molars have been found to exhibit the greatest variations in crown-root mineralization timing, which not only varies among different ethnic groups, but also in different sexes and jaws. Most of this variation may be genetic, with environmental factors exerting a lesser influence [12–17]. Thus, mineralization of third molars is not a good choice for age estimation. Gustafson [18] demonstrated that regressive dental changes in secondary dentin formation, periodontal recession, attrition, apical translucency, cementum apposition and external root resorption may be related to chronological age, which in turn could be utilized in the age estimation of adults. Subsequently, Timme et al. [19] and Olze et al. [20] developed formulae based on modified versions of Gustafson’s criteria, which did not include apical translucency and external root resorption. However, because there were too many independent factors included in the formulae, their application has been limited.

In 1995, Kvaal et al. [21] reported a method for age estimation based only on secondary dentin formation. This method was then tested by many other researchers and has proven to be quite accurate in different populations [9,22–26]. Thus, compared to other regressive dental changes, secondary dentin formation seems more strongly correlated with chronological age since it is a continual and regular process throughout life. This has also been verified by different scholars [27–30], who estimated age based on pulp/tooth ratio changes caused by secondary dentin formation.

In Cameriere’s method, canines were selected for measurement and yielded satisfactory results [27,28]. At the same time, in our previous research [31], which applied the Kvaal method to Chinese subjects, we also observed that secondary dentin formation in mandibular canines has a relatively high correlation with chronological age compared to other selected teeth [31]. Thus, in this study, we attempted to gather more details concerning the relationship between secondary dentin formation in canines and chronological age, and to verify for the first time which canines are most significantly correlated with chronological age. From this analysis, we sought to develop a new and simpler formula based on the measurements of canines according to Kvaal’s methodology.

## Materials and methods

### Samples

We randomly selected 360 orthopantomograms (OPGs) with permission from the Oral Radiological Department in the Stomatological Hospital of Xi’an Jiaotong University (Xi’an, China) from 2016 to 2018. The samples belonged to patients from northern China, whose ages ranged from 20 to 65 years (Table 1). All X-ray films had been obtained for clinical diagnosis or orthodontic treatment, without any additional financial burden on the patient. All of the selected images contained four canines, and those radiographs having (i) bad image quality, (ii) root-filled canines, (iii) any pathology in canines such as caries, periodontal and periapical inflammation, attrition, impaction and rotation, (iv) canines with any fillings, restoration, or (v) orthodontic brackets were excluded from the study. Furthermore, the selected OPGs were relabeled, and the observer was blinded to the chronological age of each sample.

**Table 1.** Age and sex distribution of the sampled northern Chinese subjects.

Age (years)	Male (*n* = 180)	Female (*n* = 180)	Total
20.00–24.99	20	20	40
25.00–29.99	20	20	40
30.00–34.99	20	20	40
35.00–39.99	20	20	40
40.00–44.99	20	20	40
45.00–49.99	20	20	40
50.00–54.99	20	20	40
55.00–59.99	20	20	40
60–65	20	20	40

### Methods

**Figure 1.**
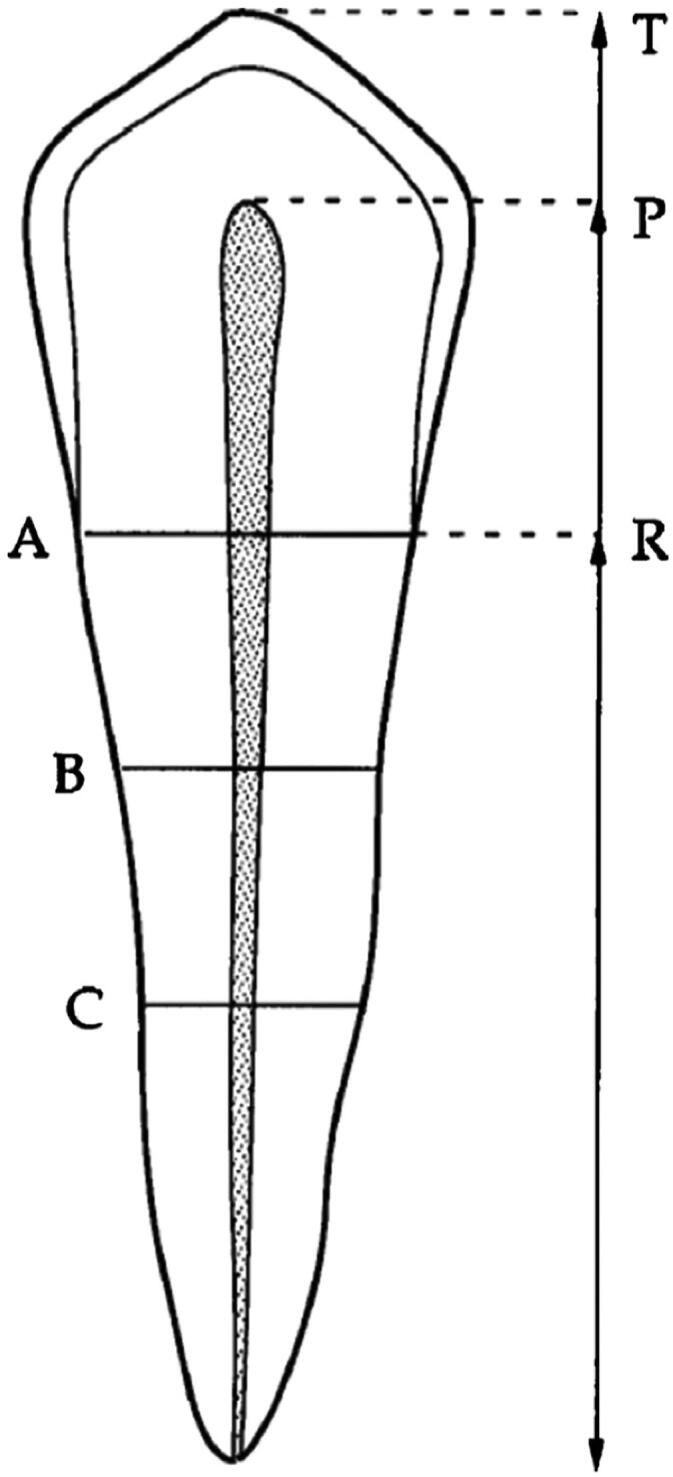
Measurements of the digital panoramic radiographs generated using Kvaal method [21]. T: maximum tooth length; P: pulp length; R: root length on the mesial surface from the enamelo-cemental junction (ECJ) to the root apex; A: level A, ECJ level; B: level B, halfway between the ECJ and mid-root level; C: level C, mid-root level.

All OPGs included in this study were measured using Adobe Photoshop CS6 (Adobe Systems, Inc., San Jose, CA, USA). In every OPG, each of the four canines was measured using Kvaal’s methodology. For each canine, six measurements were performed: maximum length of the tooth, pulp and root, as well as the root and pulp width at levels A–C (level A refers to the horizontal level at the enamelo-cemental junction (ECJ), level C refers to the horizontal level at mid-root, and level B refers to the horizontal level halfway between the ECJ and mid-root) (Figure 1). Several dental ratios were employed in order to compensate for the possible magnification and angulation of the OPGs: the length ratios of the pulp/root (P), tooth/root (T) and pulp/tooth (R), as well as the width ratios of the pulp/root at levels A–C. In addition, M is the average of all ratios mentioned above, with the exception of T; W represents the average of the width ratios at levels B and C; and L is the average of the length ratios P and R.

### Statistical analysis

In order to examine the consistency of the measurements, 40 randomly selected OPGs were evaluated a second time by two observers 2 months later. Intra-class correlation coefficients (ICCs) were then calculated to assess intra- and inter-observer consistency. This was performed to ensure that all data in this study were measured under similar conditions.

From the start, all samples included in our study were randomly divided into two groups, designated as “training” and “testing” (Table 2). During statistical analysis, which was undertaken on samples from the training group, the correlation coefficients between each ratio and chronological age were first analyzed. Next, a series of Chinese-specific formulae for age estimation were derived by applying principal component and multiple linear regression analyses. Finally, a dataset from the testing group was used to compare the accuracy among the obtained formulae. All analyses mentioned above were conducted using SPSS 18.0 statistical software (PASW Statistics for Windows, Version 18.0; SPSS Inc., Chicago, IL, USA).

**Table 2.** Distribution of the training group and test group.

Group	Male (*n* = 180)	Female (*n* = 180)	Total
Training dataset	135	135	270
Test dataset	45	45	90

## Results

The intra-observer reliability was found to range from 0.701 to 0.921, whereas the inter-observer reliability varied from 0.710 to 0.824; both of them displayed relatively high values.

The correlation coefficients between chronological age and each ratio, as well as their average values, are listed in Table 3. We found that all of these correlation coefficients were significantly lower than those determined using the Kvaal method. Overall, the length ratios exhibited more significant and higher correlations with age than the width ratios, wherein the R ratio was the highest, followed by the P ratio. The B value represents the width ratio, which also showed a relatively strong correlation with age. When taking different canines into consideration, the measurement of the left maxillary canine performed best compared with the other canines, followed by the right mandibular canine, whereas the left mandibular canine had the poorest performance, a finding that may be attributable to the overall lower correlation of mandibular canines with chronological age.

**Table 3.** Correlation coefficients between chronological age and ratios of measurements from the digital panoramic radiographs and mean of the ratios from each canine, *n* = 270.

Tooth	13	23	33	43	Maxillary canines	Mandibular canines	All canines
P	−0.108 (NS)	−0.294**	0.039 (NS)	−0.217**	−0.271**	0.011 (NS)	−0.037 (NS)
T	0.128*	−0.012 (NS)	0.084 (NS)	0.192**	0.079 (NS)	0.105 (NS)	0.117 (NS)
R	−0.268**	−0.276**	−0.375**	−0.408**	−0.344**	−0.455**	−0.458**
A	−0.078 (NS)	−0.140*	0.099 (NS)	−0.064 (NS)	−0.149*	0.057 (NS)	−0.092 (NS)
B	−0.255**	−0.346**	0.095 (NS)	−0.189**	−0.339**	−0.057 (NS)	−0.230**
C	−0.097 (NS)	−0.314**	0.086 (NS)	−0.080 (NS)	−0.153*	0.006 (NS)	−0.134*
M	−0.121*	−0.221**	0.058 (NS)	−0.152*	−0.193**	0.009 (NS)	−0.081 (NS)
W	−0.143*	−0.359**	0.103 (NS)	−0.152*	−0.238**	−0.029 (NS)	−0.192**
L	−0.205**	−0.322**	0.009 (NS)	−0.327**	−0.358**	−0.050 (NS)	−0.134*
W-L	−0.044 (NS)	0.096 (NS)	0.003 (NS)	0.197**	0.013 (NS)	0.043 (NS)	0.042 (NS)

Tooth 13: right maxillary canine; 23: left maxillary canine; 33: left mandibular canine; 43: right mandibular canine; P: ratio between length of pulp and root; T: ratio between length of tooth and root; R: ratio between length of pulp and tooth; A: ratio between width of pulp and root at enamelo-cemental junction (ECJ) (level A); B: ratio between width of pulp and root at mid-point between level A and C (level B); C: ratio between width of pulp and root at mid-root the level (level C); M: mean value of all ratios except for T; W: mean value of width ratios from levels B and C; L: mean value of the length ratios P and R; W-L: difference between W and L. NS: no significance, *P* > 0.05; *P < 0.05; **P < 0.01.

When determining the age estimation formulae, the ratios P, R and B were selected as independent variables due to their significantly high correlations with age. Principal component analysis revealed that there was only one extracted component when simplifying the estimation formulae. The main principal component for the left maxillary canine could explain more than 40% variance. Given that similar weights were attributable to the ratios of P (0.803) and R (0.794), L (the average of ratio P and R) was used in the approximation of the main principal component. However, the weights of ratio B were 0, indicating that the main principal component could not explain ratio B. Thus, both ratio B and ratio L should be used to obtain a regression formula for age estimation.

The regression formulae acquired in this study are listed in Table 4, including the formulae derived from each canine, maxillary canines, mandibular canines and all canines. The maximum coefficient of determination (*r*^2^) came from the formula derived from the left maxillary canine, whereas the minimum *r*^2^ was from the left mandibular canine formula.

**Table 4.** Regression equations for age estimation based on data in this study.

Tooth	Equation	*r*	*r* ^2^	SEE
13	Age = 61.4 + 4.1L − 111.7B	0.38	0.14	12.10
23	Age = 5.8 + 61.5L − 118.9B	0.54	0.29	11.00
33	Age = 54.6 + 4.3L − 86.1B	0.30	0.10	12.40
43	Age = 13.2 + 51.3L − 112.5B	0.41	0.17	11.90
Maxillary canines	Age = 6.9 + 67.4L − 150.8B	0.52	0.27	11.10
Mandibular canines	Age = 57.8 + 10.1L − 135.1B	0.40	0.16	12.00
All canines	Age = 52.6 + 23.8L − 172.3B	0.50	0.25	11.30

Tooth 13: right maxillary canine; 23: left maxillary canine; 33: left mandibular canine; 43: right mandibular canine; L: mean value of length ratio P and R; B: ratio between width of pulp and root at mid-point between level A and C (level B); *r*: correlation coefficients; *r*^2^: coefficient of determination; SEE: standard error of the estimate in years.

A comparison of the accuracy of the formulae obtained in this study in the testing group facilitated the determination of the average, standard deviation, minimum and maximum values of the residuals (Table 5).

**Table 5.** The statistics of residuals obtained using the Chinese-specific equation in this study.

Tooth	Equation	Residuals			
		Mean	SD	Min	Max
13	Age = 61.4 + 4.1L − 111.7B	−0.758	12.44	−28.49	23.29
23	Age = 5.8 + 61.5L − 118.9B	0.932	12.54	−25.07	35.21
33	Age = 54.6 + 4.3L − 86.1B	0.231	12.88	−26.14	24.77
43	Age = 13.2 + 51.3L − 112.5B	−0.680	12.25	−35.65	22.63
Maxillary canines	Age = 6.9 + 67.4L − 150.8B	0.614	12.13	−26.11	25.40
Mandibular canines	Age = 57.8 + 10.1L − 135.1B	−0.100	12.29	−30.01	22.09
All canines	Age = 52.6 + 23.8L − 172.3B	0.010	11.81	−30.74	22.93

Tooth 13: right maxillary canine; 23: left maxillary canine; 33: left mandibular canine; 43: right mandibular canine; L: mean value of length ratio P and R; B: ratio between width of pulp and root at mid-point between level A and C (level B); SD: standard deviation; Min: minimum residuals; Max: maximum residuals.

## Discussion

Many published studies have confirmed the use of secondary dentin formation in age estimation since it is a continuous and universal process, influenced only by tooth decay or severe attrition [21,22,28]. In our previous study [31], when applying the Kvaal method to Chinese subjects, we observed that the mandibular canines exhibited the highest correlation with chronological age. However, in some papers that adopted the Kvaal method for age estimation of different populations, the results showed discrepancies; specifically, mandibular canines showed relatively good correlation with age in Turkish [24] and Korean [9] individuals, but not in Western Australian populations [26]. Tooth attrition is related to diet, habits and culture [32,33], which may contribute to the diverse correlations between canine and age among different populations. Thus, it has been recommended that age estimation formulae based on the formation of secondary dentin should be population-specific.

Cameriere et al. [27] previously developed an alternative method for age estimation, a technique that was completely different from the Kvaal method but was also based on the formation of secondary dentin. In Cameriere’s methodology, all canines were selected for measurement. There are two possible reasons for this approach. First, canines have the longest functional survival rate in the mouth due to their long roots. Second, since canines have long roots and distinct pulp margins, they can be readily measured [27,28].

Unlike Cameriere’s method, however, which draws the outline of canines and their pulps using a specific computer-aided drafting program, the Kvaal method is relatively simple and convenient since it only requires the measurements of several linear indices. Thus, the present study verified the appropriate utilization of canines for age estimation based on the Kvaal method.

Nevertheless, which canine has the highest correlation with chronological age remains controversial. In 1968, Philippas and Applebaum [34] suggested that the size and shape of the pulp cavity are similar between the upper and lower canines. According to Cameriere’s study, age estimation formulae independently derived from the upper and lower canines had similar residual standard errors of approximately 5.44 years, and the coefficients of determination were 0.86, indicating that there were no significant differences between the upper and lower canines when estimating age based on secondary dentin [27]. In our study, however, the correlation between chronological age and mandibular canines was significantly worse than that of maxillary canines. Furthermore, the coefficient of determination and standard error values of the formula derived from mandibular canines were not as good as those of maxillary canines. These differences may be due to the poor correlation between left mandibular canines and chronological age. Azevedo et al. [35] recommended that only maxillary canines be selected for age estimation utilizing Cameriere’s method. Although the presence of two roots or at least two canals in mandibular canines were uncommon, they were not readily perceived in X-rays. In another study [29], however, researchers only selected mandibular canines for age estimation, since they believed that the periapical films of mandibular canines are more precise than those of maxillary canines as it is easier to properly situate the rigid sensor parallel to the tooth in the mandibular lingual sulcus when taking periapical images [29]. In addition, mandibular canines were consistently observed in all age groups in this study.

The previous study based on the Kvaal method did not observe any significant differences between teeth from the left and the right side of jaw [21]. Therefore, all subsequent studies using the Kvaal method assumed that the measurements of bilateral teeth were consistent. In actuality, only 20 samples were included in the preliminary study conducted by Kvaal et al. [21]. In addition, variations in diet and eating habits such as unilateral mastication might contribute to different physiological changes in teeth in different populations. Thus, we suggest reconsidering whether the rate of secondary dentin formation is the same in the bilateral canines of Chinese subjects. According to our study, the formation of secondary dentin exhibits significant differences between maxillary and mandibular canines when estimating age, although no obvious trend as to which side is better for age estimation was observed, since the left maxillary canine seems better correlated with age than the right, while the opposite result was found for the mandibular canines.

In our study, the highest coefficient of determination (0.29) was observed using the regression equation that was developed for the left maxillary canine, which had an 11-year standard error. The coefficient for maxillary canines (0.27) ranked second, followed by the coefficient for all canines (0.25). All of these values were higher than the highest coefficient of determination from the Chinese-specific equation (0.23) that was derived from six different types of teeth (maxillary central and lateral incisors, second premolars, mandibular lateral incisors, canines and first premolars). These results indicate that when applying the Kvaal method for age estimation to the northern Chinese population, canines demonstrated the advantage of being more accurate than other teeth. Furthermore, when verifying the accuracy of the associated formulae *via* the testing group dataset in the present study, we found that the mean value and standard error of the residuals for all canines performed best, with values of 0.010 and 11.81 years, respectively. These are significantly smaller than the minimum mean value and standard error of the residuals in our previous study [31], derived from the Chinese-specific formulae based on six different types of teeth, which were 3.4 and 11.9 years, respectively. We also observed that all of the mean values and standard errors of the residuals in the present study were smaller than the minimum values in our previous study, further demonstrating the superiority of canines in age estimation when using the Kvaal method. However, since our results were only based on the northern Chinese population, additional samples from different geographic regions and populations are required in order to further confirm whether canines are a good choice for age estimation when using the Kvaal method.

## Conclusion

Canines were discovered to play an important role in the age estimation of the northern Chinese population. The correlation between maxillary canines and chronological age is stronger than that of mandibular canines, although no distinct trend in terms of which side is better correlated with age was established. Going forward, we recommend the analysis of additional samples from different geographical regions and populations in order to further verify the importance of canines in age estimation.
